# Supplementary material for: Spatial contextual effects in primary visual cortex limit feature representation under crowding
Source: Nat Commun. 2020 Apr 3;11:1687. doi: 10.1038/s41467-020-15386-7 (PMC7125172; doi:10.1038/s41467-020-15386-7)
Supplement: Supplementary file 2 — Reporting Summary [file 41467_2020_15386_MOESM2_ESM.pdf]

nature research

Corresponding author(s): Kohn, Henry

Last updated by author(s): Mar 2, 2020

Reporting Summary

Nature Research wishes to improve the reproducibility of the work that we publish. This form provides structure for consistency and transparency in reporting. For further information on Nature Research policies, see Authors & Referees and the Editorial Policy Checklist .

Statistics

For all statistical analyses, confirm that the following items are present in the figure legend, table legend, main text, or Methods section.

n/a	Confirmed	
☐	☒	The exact sample size (*n*) for each experimental group/condition, given as a discrete number and unit of measurement
☐	☒	A statement on whether measurements were taken from distinct samples or whether the same sample was measured repeatedly
☐	☒	The statistical test(s) used AND whether they are one- or two-sided<br>*Only common tests should be described solely by name; describe more complex techniques in the Methods section.*
☐	☒	A description of all covariates tested
☒	☐	A description of any assumptions or corrections, such as tests of normality and adjustment for multiple comparisons
☐	☒	A full description of the statistical parameters including central tendency (e.g. means) or other basic estimates (e.g. regression coefficient) AND variation (e.g. standard deviation) or associated estimates of uncertainty (e.g. confidence intervals)
☐	☒	For null hypothesis testing, the test statistic (e.g. *F*, *t*, *r*) with confidence intervals, effect sizes, degrees of freedom and *P* value noted<br>*Give P values as exact values whenever suitable.*
☒	☐	For Bayesian analysis, information on the choice of priors and Markov chain Monte Carlo settings
☒	☐	For hierarchical and complex designs, identification of the appropriate level for tests and full reporting of outcomes
☒	☐	Estimates of effect sizes (e.g. Cohen's *d*, Pearson's *r*), indicating how they were calculated

Our web collection on statistics for biologists contains articles on many of the points above.

Software and code

Policy information about availability of computer code

Data collection	Human perceptual data were collected using Psychtoolbox 3, a common open source library for perceptual experimentation. Monkey perceptual data were collected using EXPO (https://sites.google.com/a/nyu.edu/expo/). Spiking activity was recorded using commercial data acquisition software (Blackrock, UT). Spiking data were sorted using Spike2 v3 (Plexon, TX).
Data analysis	Data were analyzed using custom scripts in Matlab (R2014a; Mathworks, MA). All code is available upon request.

For manuscripts utilizing custom algorithms or software that are central to the research but not yet described in published literature, software must be made available to editors/reviewers. We strongly encourage code deposition in a community repository (e.g. GitHub). See the Nature Research guidelines for submitting code & software for further information.

Data

Policy information about availability of data

All manuscripts must include a data availability statement. This statement should provide the following information, where applicable:

- Accession codes, unique identifiers, or web links for publicly available datasets
- A list of figures that have associated raw data
- A description of any restrictions on data availability

All figures were generated using raw data collected for this study. Data are available upon request. Source data is provided for Figures 1 and 3-8 as a source data file.

Field-specific reporting

Please select the one below that is the best fit for your research. If you are not sure, read the appropriate sections before making your selection.

Life sciences study design

All studies must disclose on these points even when the disclosure is negative.

Sample size	Sample size for perceptual experiments was 2-3 human subjects and 3 monkeys. Sample size for the neurophysiological recordings was 8 array implants in 6 animals. These sample sizes are consistent with previous neurophysiological studies of early visual processing.
Data exclusions	In monkey perceptual experiments, trials were excluded if subjects gaze exited the fixation window. For neuronal data, we excluded neurons which did not respond to visual targets with a rate that was greater than 0.5 sp/s and 2 s.d. above the mean spontaneous firing rate.
Replication	Each perceptual result was obtained in at least two human/monkey subjects. Neurophysiological results were based on recordings from several hundred neurons (depending on analysis) recorded from 8 multielectrode array implants in 6 different animals.
Randomization	Each subject (human subject, monkey, or neuron) provided both control (target alone) and experimental condition (target and distractors) data. Conditions were presented to each subject in a randomized manner.
Blinding	Investigators were not blinded during data collection or analysis. However, all conditions (responses to targets vs targets and distractors) were processed by the same code and pipeline.

Reporting for specific materials, systems and methods

We require information from authors about some types of materials, experimental systems and methods used in many studies. Here, indicate whether each material, system or method listed is relevant to your study. If you are not sure if a list item applies to your research, read the appropriate section before selecting a response.

Materials & experimental systems

n/a	Involved in the study	
☒	☐	Antibodies
☒	☐	Eukaryotic cell lines
☒	☐	Palaeontology
☐	☒	Animals and other organisms
☐	☒	Human research participants
☒	☐	Clinical data

Methods

n/a	Involved in the study	
☒	☐	ChIP-seq
☒	☐	Flow cytometry
☒	☐	MRI-based neuroimaging

Animals and other organisms

Policy information about studies involving animals; ARRIVE guidelines recommended for reporting animal research

Laboratory animals	Macaca fascicularis, male, 3-9 years
Wild animals	Study did not involve wild animals. Animals were purchased from a commercial vendor.
Field-collected samples	N/A
Ethics oversight	All animal work was performed under protocols approved by the Institutional Animal Care and Use Committee of the Albert Einstein College of Medicine

Note that full information on the approval of the study protocol must also be provided in the manuscript.

Human research participants

Policy information about studies involving human research participants

Population characteristics	Participants were 3 male adults, age 25-50, with normal or corrected to normal vision.
Recruitment	Participants were recruited on campus; no exclusion criteria were used to screen participants.
Ethics oversight	All human work was performed under a protocol approved by the Institutional Review Board of the Albert Einstein College of Medicine.

Note that full information on the approval of the study protocol must also be provided in the manuscript.
